# Label-free paper-based electrochemical aptasensor with tunable selectivity for assessing neurotransmitter imbalance in Alzheimer’s disease

**DOI:** 10.1007/s00604-026-08100-9

**Published:** 2026-05-07

**Authors:** Silvia Dortez, Miriam Chávez, Ana Montero-Calle, Rodrigo Barderas, Marta Pacheco, Alberto Escarpa

**Affiliations:** 1 Department of Analytical Chemistry, Physical Chemistry and Chemical Engineering, University of Alcala, Alcala de Henares, Madrid 28802 Spain; 2 Department of Physical Chemistry and Applied Thermodynamics, Institute of Chemistry for Energy and Environment, University of Cordoba, Campus Rabanales, Ed. Marie Curie, Córdoba, E- 14014 Spain; 3 Chronic Disease Programme, UFIEC, Institute of Health Carlos III, Majadahonda, Madrid 28220 Spain; 4 CIBER of Frailty and Healthy Aging, CIBERFES, Madrid, 28029 Spain; 5 Chemical Research Institute “Andrés M. Del Río” (IQAR), University of Alcala, Alcala de Henares, Madrid 28802 Spain

**Keywords:** Aptassay, Electrochemical paper-based analytical devices, Neurotransmitters, Dopaminergic and serotonergic dysfunctions, Neurodegenerative diseases

## Abstract

A paper-based electrochemical aptasensor (PEA) is reported for the simultaneous detection of extracellular dopamine (DA) and serotonin (SE) in human brain samples, enabling the assessment of neurotransmitter imbalances associated with Alzheimer’s disease (AD). The PEA integrates a dual-selectivity strategy that combines aptamer-mediated molecular recognition with potential-resolved electrochemical neurotransmitters discrimination, allowing reliable differentiation between healthy and AD-diagnosed left prefrontal cortex tissues based on altered dopaminergic and serotonergic levels. Thiolated aptamers immobilized onto gold nanoparticles (AuNPs) at the paper electrode interface provide selective neurotransmitter biorecognition, while the distinct oxidation potentials of DA (+ 0.10 V) and SE (+ 0.25 V *vs*. Ag/AgCl) enable their label-free, simultaneous quantification on a single disposable electrode. The PEA read biologically meaningful concentration shifts across a clinically relevant dynamic range, spanning levels characteristic of healthy tissue to those observed in advanced pathological stages. The PEA approach highlights its potential for liquid biopsy–oriented neurochemical monitoring and supports its application in multiplexed biosensing within organ-on-a-chip systems, enabling early diagnostic strategies for neurodegenerative diseases.

**Graphical Abstract:**

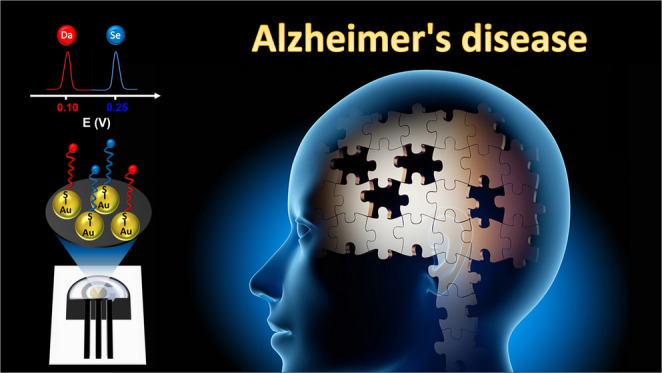

**Supplementary Information:**

The online version contains supplementary material available at 10.1007/s00604-026-08100-9.

## Introduction

Paper has emerged as an attractive substrate for analytical devices due to its low cost, wide availability, flexibility, porosity, hydrophilicity, and environmental sustainability [1]. In electrochemical applications, it provides a non-reactive background and supports the deposition of conductive inks, enabling its transformation into an electroactive platform [2, 3]. Within this context, electrochemical paper-based analytical devices (ePADs) offer significant advantages, including portability, disposability, rapid response, high sensitivity, and low sample consumption [4]. Notably, ePADs exhibit excellent customizability and a high degree of versatility in electrode design, allowing flexible configurations of electrochemical cells tailored to specific analytical needs [5–7]. These features, together with their capability for multiplexed analysis, make ePADs particularly suitable for decentralized and *point-of-care* (POC)/*point-of-need* applications [8].

Aptamers, single-stranded DNA or RNA oligonucleotides have emerged as highly specific molecular recognition elements in biosensors [9]. These synthetic ligands are generated through an iterative selection process known as Systematic Evolution of Ligands by Exponential Enrichment (SELEX) and exhibit high binding affinities for a broad range of target molecules, including small molecules, proteins, and cells [10, 11]. Compared to antibody-based recognition systems, aptamers offer several advantages: they are non-immunogenic, chemically stable, easily synthesized, and cost-effective [12]. Their small size and structural flexibility allow for diverse biosensing strategies, including conformational changes upon target binding that can be readily transduced into electrochemical signals. Additionally, aptamers retain their functionality after denaturation, enabling long-term storage and repeated use in analytical applications. These attributes make aptamers ideal candidates for use in ePADs, where selectivity and stability are crucial [13, 14].

Gold nanoparticles (AuNPs) have been widely used in biosensing due to their unique optical and electronic properties, excellent biocompatibility, and well‑established surface chemistry [15, 16]. In particular, citrate‑stabilized AuNPs synthesized via the classical Turkevich method exhibit a stable surface suitable for functionalization [17], and their strong affinity for thiolated molecules enables the formation of robust Au–S covalent bonds [18]. This makes AuNPs ideal anchoring platforms for aptamers, ensuring stable orientation, high surface coverage, and efficient biofunctionalization in electrochemical interfaces.

On the other hand, neurotransmitters such as dopamine (DA) and serotonin (SE) play essential roles in regulating physiological and neurological processes, including mood, cognition, and motor control [19]. Dysregulation of these neurotransmitters has been implicated in neurological and psychiatric disorders, including Alzheimer’s disease (AD) [20]. Beyond its classical hallmarks, amyloid-beta plaques and tau neurofibrillary tangles, AD is associated with early neurotransmitter imbalances [21]. Importantly, early alterations in dopaminergic and serotonergic signaling have been increasingly linked to neuropsychiatric and cognitive symptoms that precede overt memory decline in AD. Dopaminergic dysfunction in AD has been the subject of numerous studies, highlighting alterations in both DA synthesis and release [22, 23]. Key regions involved in this process include the ventral tegmental area and its connections to cortical and subcortical areas, such as the hippocampus, prefrontal cortex, and basal ganglia. These dopaminergic alterations correlate with early neuropsychiatric symptoms, such as apathy and depression, which often precede the cognitive decline characteristic of AD. Post-mortem studies have observed a reduction in dopaminergic activity and changes in receptor density, reinforcing the role of DA in disease progression [24, 25]. Similarly, the serotonergic system is significantly disrupted in AD, with documented decreases in both SE levels and receptor density, particularly in the temporal and frontal cortices. Functional impairments in SE signalling have been correlated with neuropsychiatric symptoms such as aggression, depression, and anxiety [19, 26]. Notably, selective SE reuptake inhibitors have demonstrated potential in alleviating behavioural and cognitive symptoms in AD patients, highlighting the therapeutic relevance of targeting SE dysregulation [27, 28]. Given the growing recognition of neurotransmitter alterations as early biomarkers of AD, the development of reliable sensing platforms for multiplex detection of DA and SE has become useful and electrochemical sensors are particularly attractive for this purpose due to their high sensitivity, portability, low cost, and suitability for POC analysis [29, 30].

In this context, the integration of aptamer-based molecular recognition with ePADs, enabling he development of paper‑based electrochemical aptasensors (PEAs), which could conceptually offer a promising approach for (real-time) monitoring of neurotransmitter dynamics in both research and clinical settings.

Here, we report the development of a disposable PEA for the simultaneous determination of extracellular SE and DA in brain samples from patients with AD and healthy individuals. The paper-based architecture provides a low-cost, scalable, and easily implementable platform well suited for portable and POC neurochemical analysis and multiplexed biosensing in organ-(brain)-on-chip systems. The PEA is based on a selectivity-tuning strategy that integrates aptamer-mediated molecular recognition with potential-resolved electrochemical discrimination. Together, these complementary selectivity mechanisms ensure accurate and interference‑resistant neurotransmitter profiling in complex brain tissue extracts. Following a well-established chemistry, thiolated aptamers immobilized onto AuNPs ensure selective neurotransmitter capture, while the distinct oxidation potentials of DA and SE enable their label-free, simultaneous quantification on a single electrode. This dual-selectivity approach allows reliable neurotransmitter profiling in complex left prefrontal cortex tissues without the need for labeling strategies, exploiting the intrinsic electroactivity of both analytes. By enabling assessment of dopaminergic and serotonergic imbalances associated with AD progression, the proposed PEA establishes a scalable and low-cost analytical platform with strong potential for translational research, early diagnostic applications, and future integration into organ-on-chip and *point-of-care* testing systems.

## Experimental

### Reagents, materials, and samples

Uric acid, glucose, serotonin, dopamine, norepinephrine potassium hexacyanoferrate (II) trihydrate, tetrachloroauric (III) acid trihydrate, 1-mercaptohexanol, bovine serum albumin (BSA), 1,6-hexanedithiol, sodium citrate, and silver/silver chloride (60/40) paste were purchased from Merck (Darmstadt, Germany). Potassium chloride was purchased from Scharlau (Barcelona, Spain). Potassium hexacyanoferrate (III) was purchased from PanReac (Barcelona, Spain). Carbon paste (BG04) was purchased from SunChemical (New Jersey, USA). Phosphate buffered saline (PBS) 0.1 M pH 7.2, ascorbic acid, and sodium chloride were acquired from Thermo Fisher Scientific (USA). Thiol modified aptamers for DA (5’-SH-GTCTCTGTGTGCGCCAGAGAACACTGGGGCAGATATGGGCCAGCACAGAATGAGGCCC-3’) and SE (5’-SH-CTCTCGGGACGACTGGTAGGCAGATAGGGGAAGCTGATTCGATGCGTGGGTCGTCCC-3’) were obtained from Biomers (Germany) and the secondary structure and thermodynamic parameters shown in Figure S1 (see Supplementary Information file) were predicted with the computer program of modelling MFold.

All reagents and solvents were of analytical grade. All solutions were prepared in deionized ultrapure water (Merck Millipore, Darmstadt, Germany).

Whatman Chromatography Paper 1 CHR was purchased from Merck (Darmstadt, Germany). The waterproof marker pen Lumocolor^®^ permanent CD/DVD/BD 310 was purchased from Staedtler (Nuremberg, Germany). Tesa 4024 clear packing tape and adhesive vinyl sheets were purchased from Amazon (Spain).

Pseudonymized samples of protein extracts of left prefrontal cortex frozen tissue (0.25 µg µL^−1^) from patients with AD and healthy individuals were prepared at the Instituto de Salud Carlos III (Madrid, Spain), following established protocols [31] to simultaneously assess the presence of DA and SE in healthy individuals and AD patients. All samples were stored at − 80 °C until used. The Institutional Ethical Review Board of the Spanish Research Center for Neurological Diseases Foundation (CIEN) and the Instituto de Salud Carlos III approved this study on proteomics analysis and biomarker discovery of AD (CEI PI 49 and CEI PI 74).

### Instrumentation

#### Design

AutoCAD 2018 (Autodesk, Student Version) was used to design all ePAD components. A desktop cutting plotter (Silhouette Cameo 3, Silhouette) was employed to transfer the electrode design onto a sheet of filter paper (Whatman 1 CHR).

#### Electrochemical measurements

Potentiostat Autolab PGSTAT101, including a module to record electrochemical impedance measurements (Methrom-Autolab, the Netherlands), was used for all the electrochemical measurements, controlled by using the software Nova 2.1.5. For differential pulse voltammetry (DPV) signals, when necessary, baseline correction was performed by linear background subtraction using the OriginPro 8.5 software prior to peak current extraction.

#### Gold nanoparticle characterization and zeta-potential measurements

The size distribution of the AuNPs was determined by transmission electron microscopy (TEM) using a JEOL JEM‑1400 microscope operated at an accelerating voltage of 80–120 kV. TEM images were analyzed using Image‑Pro Plus software to determine particle size and size distribution. Additionally, the quantification and optical size estimation of the synthesized AuNPs were performed by ultraviolet-visible (UV-vis) spectroscopy using a microplate reader (Synergy HTX, BioTek), monitoring the surface plasmon resonance band at 520 nm. The hydrodynamic size and zeta potential were measured by dynamic light scattering (DLS) using a Malvern Zetasizer Nano ZSP, equipped with a 633 nm He-Ne laser.

#### Sample preparation

An incubator (Eppendorf ThermoMixer C, Hamburg, Germany) was used for the aptamers reconditioning, an oven for ink curing (Conterm J.P. Selecta, Barcelona, Spain), and a hot air dryer to assist vinyl removed (Braun Cosmo 1000, Basingstoke, United Kingdom).

### Procedures

#### ePAD design and fabrication

Whatman 1 CHR was used as the filter paper because it is hydrophilic, homogeneous, biocompatible, reproducible, and cheap, among others.

Figure S2 shows the schematic procedure of the ePAD fabrication, which consists of three steps:

(i) To draw the pattern of the electrode, AutoCAD software was used (Figure S2A, top). This pattern was transferred to a piece of filter paper (Whatman 1 CHR) using a cutter plot, in which the blade was replaced by a waterproof marker pen. In this process, the waterproof marker pen ink penetrates the paper to form the hydrophobic walls on the filter paper (which acts as a hydrophilic area), allowing the aqueous liquids flowing along the edges of marker pen wall, being confined within a hydrophilic area, serving as evidence of hydrophobicity of the walls, well-created with the waterproof marker pen on both sides of the device. Additionally, a thicker waterproof marker was used to mark the part that will delimit the electrochemical cell with its connectors. This step was crucial to define the testing area and confine the solution in the delimited electrochemical cell area, thus avoiding its diffusion towards the electrical connections and affecting the readout (Figure S2B, top) [5, 32, 33].
(ii) Then, in the same way as in (i), the three-electrode pattern, consisting of Ag/AgCl and carbon electrodes, was transferred to an adhesive vinyl sheet using a blade as an electrode cutter (Figure S2A, middle and bottom, respectively). After the hydrophilic area of filter paper was surrounded by a hydrophobic wall, these Ag/AgCl and carbon adhesive vinyl patterns were stuck on the piece of filter paper where the electrode was drawn (Figure S2B, middle and bottom, respectively). The stencil-printing process was then performed manually onto the hydrophilic semicircular electrochemical detection area (Figure S2C), using a squeegee and two adhesive vinyl masks (one for the Ag/AgCl pattern and the other for the carbon pattern). First, the adhesive vinyl mask with the Ag/AgCl pattern was pasted on the filter paper and a layer of Ag/AgCl ink was applied using a flat squeegee to create the reference electrode and the electrical connections (Figure S2C, top). The stencil-printed devices were then cured in an oven at 90 °C for 30 min. After curing, the adhesive vinyl mask was removed with the assistance of a hot air dryer. Second, the adhesive vinyl mask with the carbon pattern was pasted on the filter paper and a layer of carbon ink was applied in the same way using a flat squeegee to create the working and counter electrodes (Figure S2C, bottom). The devices were allowed to cure at 120 °C for 30 min in an oven and after that time, the adhesive vinyl mask was removed with the help of a hot air dryer. Thermal curing was essential to ensure the printed ink stable for electrochemical measurements. Once the fabrication was completed, each electrode was individually cut and the backside of the printing surface was covered with clear packing tape (without covering electrical connections) to prevent the solution from leaking out underneath of the electrochemical cell (Figure S2D).
(iii) In order to facilitate the anchoring of the thiolated aptamers of DA and SE on the surface of the working electrode (WE), the electrode was carefully modified by drop casting with 8 µL of 10 nM AuNPs onto the WE, allowing them to dry completely at room temperature, and washing three times with 30 µL of PBS (0.1 M, pH 7.2) (Figure S2E). Finally, they were ready for the modification with thiolated aptamers. AuNPs were used to modify the WE surface, as their anchoring with thiolated compounds is well-known through the formation of Au–S covalent bonds [18].

The electrodes consisted of a hydrophilic electrochemical semicircular detection reservoir (electrochemical cell of 13 mm diameter). The electrochemical cell consisted of three 19 mm x 1 mm (length x width) rectangles separated by 1.5 mm, serving as electrical connectors. The reference electrode was designed as an arc of 3 mm x 1 mm (length x width), the WE was a circle of 4 mm diameter, and the counter electrode was an arc of 12 mm x 1 mm (length x width). The protective rectangle for connections was 5 mm x 13 mm (length x width). The entire design of the electrodes was reported in Figure S3.

In a single sheet, 40 devices were drawn and stencil-printed. Consequently, each electrode was cut to obtain disposable devices after use.

### Gold nanoparticles synthesis

WEs of the ePADs were modified using spherical AuNPs, which were synthesized following the classic Turkevich synthesis method [17]. Initially, 50 mL of a 1 mM HAuCl₄ solution was prepared and brought to a boil with minimal stirring. Next, 5 mL of a 38.8 mM sodium citrate solution was added while stirring, leading to a colour change from pale yellow to deep red, indicating nanoparticle formation, and the mixture was boiled for 15 min. The solution was then allowed to cool at room temperature. The AuNPs were stored at 4 °C until use, and they were sonicated in an ultrasonic bath for 5 min before use.

### Modification of ePAD with DA and SE thiolated aptamers

Prior to electrode functionalization to obtain the PEAs with thiolated DA and SE aptamers, the aptamers were reconstituted in PBS (0.1 M and pH 7.2) by heating the DNA strands (90 °C, 5 min) followed by cooling (4 °C, 5 min) for further use. The fabricated and AuNPs-modified electrodes were modified by drop casting with 15 µL of a thiolated DA and SE aptamer solution (3.7 µM each) onto the WE surface. This yields a self-assembled monolayer of aptamers on the AuNPs electrode surface via Au–thiol binding. The devices were then left to dry at room temperature for 1 h. After drying, they were rinsed three times with 20 µL of PBS (0.1 M, pH 7.2) to remove any unbound aptamers. To minimize nonspecific binding/adsorption, the electrode surface was blocked by adding 15 µL of 7 mM 1-mercaptohexanol (MCH) in PBS (0.1 M, pH 7.2), until dryness (1 h at room temperature), followed by an additional rinse with 20 µL of PBS (0.1 M, pH 7.2).

### Electrochemical measurements

After functionalizing the AuNPs-electrode with thiolated DA and SE aptamers and applying the blocking agent, electrochemical detection was performed using DPV. 15 µL (3.75 µg) of protein extracts sample was dropped onto the WE and incubated for 1 h at room temperature. After incubation, the surface was rinsed three times with 20 µL of PBS (0.1 M, pH 7.2). Subsequently, 50 µL of PBS (0.1 M, pH 7.2) were added, and DPV was performed with a potential range from − 0.1 V to + 0.6 V, with a step potential of 0.005 V, an amplitude of 0.025 V, and a modulation time of 0.05 s. Control (DA and SE thiolated aptamers, plus the blocking agent) and blank (0.1 M PBS pH 7.2) samples were also prepared and analyzed in the same way.

The different stages during the PEA fabrication (bare electrode, AuNPs-electrode, aptamers-AuNPs-electrode, and MCH-aptamers-AuNPs-electrode) were characterized by electrochemical impedance spectroscopy (EIS). EIS measurements were recorded in the presence of 50 µL of 5 mM K_4_Fe(CN)_6_/K_3_Fe(CN)_6_ in 0.1 M KCl electrolyte as redox probe, using E_p_ = 0.1 V (*vs*. Ag/AgCl), 10 mV amplitude, and frequencies range from 10^5^ to 0.01 Hz. In the same medium, Cyclic voltammetry (CV) was performed to confirm the formation of a self-assembled monolayer of aptamers on the AuNPs-modified electrode via Au–thiol bonding, using a potential window from − 0.5 V to + 1.0 V and a scan rate of 0.1 V s^−1^.

The electroactive surface area (A_e_) of the WE was characterized by CV using a reversible redox probe and the Randles–Sevcik equation. K_4_Fe(CN)_6_/K_3_Fe(CN)_6_ was selected as a surface-sensitivity redox probe and the electrode surface area was calculated by the slope of i_p_ *vs*. v^1/2^ plot. With this purpose, cyclic voltammograms of bare electrode and after drop casting modification using 10 nM AuNPs-colloidal suspension (AuNPs-electrode) were recorded from − 0.5 V to + 0.7 V at different scan rates (from *v* = 0.01 to *v* = 0.1 V s^−1^) using 50 µL of 5 mM K_4_Fe(CN)_6_/K_3_Fe(CN)_6_ in 0.1 M KCl electrolyte (*n* = 1 and D_0_ = 7.6 × 10^− 6^ cm^2^ s^− 1^).

All measurements were performed in triplicate using independent PEAs.

## Results and discussion

### Design of a paper-based electrochemical aptasensor: tuning selectivity

**Fig. 1:**
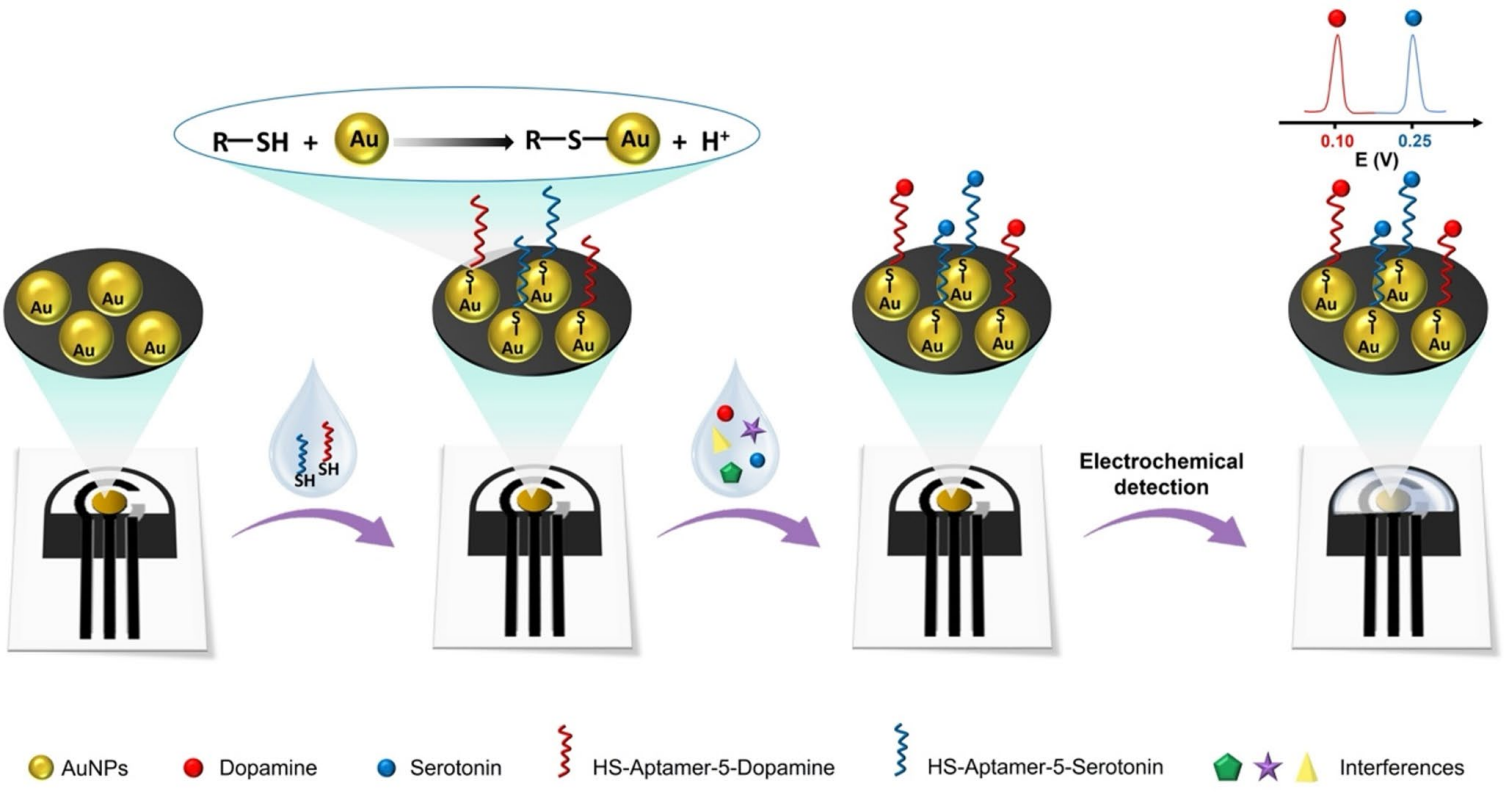
Schematics of label-free PEAs for dual electrochemical detection of SE and DA (for electrode dimensions see Figure S2)

Figure 1 illustrates the proposed design of PEA for the simultaneous detection of extracellular levels of DA and SE in complex brain tissue samples from AD patients. The strategy integrates two complementary mechanisms: (i) molecular neurotransmitter biorecognition, provided by the thiolated aptamers for DA and SE anchored onto AuNPs on the WE surface, and (ii) electrochemical neurotransmitter discrimination, exploiting their distinct anodic peaks recorded by DPV (E_p_ (DA) = + 0.10 V and E_p_ (SE) = + 0.25 V *vs*. Ag/AgCl). By combining a target‑specific interaction with the intrinsic electrochemical signatures of each neurotransmitter, the system enables selective biorecognition of DA and SE while simultaneously resolving their oxidation signals on a single electrode. This dual‑selectivity configuration enables the label‑free, direct, and reliable determination of both neurotransmitters even in the presence of electroactive interferents.

**Fig. 2:**
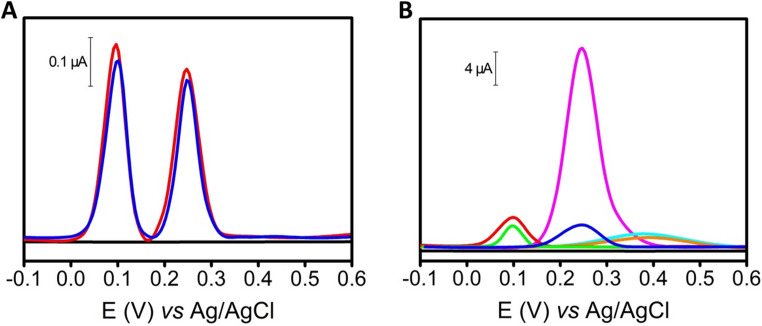
**(A)** PEAs (MCH-aptamers-AuNPs-electrode). Differential pulse voltammograms recorded in PBS (0.1 M, pH 7.2): blank (black line); a mixture of DA and SE (25 µM each) (red line); and a mixture of DA and SE (25 µM each) in the presence of potential interferents—UA, AA, Gluc, and NE (100 µM each) (blue line). **(B)** ePADs (AuNPs-modified electrodes without aptamer functionalization). Differential pulse voltammograms of individual analytes (100 µM each) recorded in PBS (0.1 M, pH 7.2): blank (black line); DA (green), SE (blue), UA (purple), AA (cyan), Gluc (orange), and NE (red). DPV parameters: start potential − 0.1 V, end potential + 0.6 V, step potential 0.005 V, amplitude 0.025 V, modulation time 0.05 s; background signal corrected (*n* = 3). Sample volume: 50 µL

The selectivity of the PEA was assessed by DPV in solution containing DA and SE (pH = 7.2; 25 µM each), and in a mixture including common electroactive interferents (uric acid (UA), ascorbic acid (AA), glucose (Gluc), and norepinephrine (NE), 100 µM each), some of which are naturally present in brain tissue, including the prefrontal cortex [34]. When using AuNPs-aptamers-electrodes, well-defined peaks for DA (+ 0.10 V) and SE (+ 0.25 V *vs*. Ag/AgCl) were observed (Fig. 2A), demonstrating that the PEAs provide excellent selectivity even in complex samples. This is further enhanced by surface passivation with MCH, which minimizes non-specific adsorption and enables selective neurotransmitter detection.

In contrast, as shown in Fig. 2B, ePADs lacking aptamers display poorly resolved anodic signals for the neurotransmitters in the presence of interferents. This behavior arises from the similar oxidation potentials of these compounds, which hinders their selective detection. Indeed, both analytes and interferents remain non-specifically adsorbed on the AuNPs surface, leading to broad and overlapping oxidation signals. Importantly, while ePADs (AuNPs-modified electrodes without aptamer functionalization) are prone to non-specific adsorption, the aptamer-modified surface in the PEA effectively suppresses this effect. These results underscore the critical role of aptamer-based molecular recognition in achieving high selectivity and reliable potential detection of DA and SE in brain samples.

### Characterization and optimization of a paper-based electrochemical aptasensor

Then, PEAs optimization for simultaneous and selective DA and SE detection was investigated. The WE was modified with AuNPs, creating a layer that enables the immobilization of the thiolated aptamers for the high-selective electrochemical detection of DA and SE via Au–S covalent bonds [18]. Prior to electrode modification, the size and colloidal properties of the pristine AuNPs were thoroughly characterized. TEM revealed the formation of quasi-spherical nanoparticles with an average core diameter of 14 ± 3 nm (Figure S4A and B, blue bar). UV–vis spectroscopy displayed a well-defined surface plasmon resonance band centered at 521 nm, corresponding to a core diameter of approximately 15 ± 1 nm (Figure S4B, pink bar) [35, 36]. DLS measurements yielded a larger hydrodynamic diameter of 36 ± 1 nm (intensity-weighted peak), as expected due to solvation effects and the contribution of the diffuse electrical layer in aqueous medium (Figure S4B, green bar). The agreement between UV–vis spectroscopy and TEM data, together with the consistent increase observed in the hydrodynamic diameter under solution conditions, supports the reliability of the estimated AuNP core size. Additionally, the ζ-potential of the pristine AuNPs was − 39 ± 3 mV, indicating good colloidal stability. This relatively high negative value is consistent with the presence of citrate ions adsorbed on the nanoparticle surface, which provide electrostatic repulsion and effectively prevent aggregation, confirming the stability of the AuNPs dispersion under the experimental conditions.

Subsequently, the paper-based electrode surface was modified with the previously characterized AuNPs, and the A_e_ was estimated and determined using the Randles-Sevcik equation using [Fe(CN)_6_]^3−/4−^ as probe. Bare electrode showed an apparent A_e_ of 0.17 ± 0.01 cm^2^. Upon modification, via drop casting using 10 nM citrate-stabilized AuNPs solution, the area arises to 0.21 ± 0.01 cm^2^ which leads to a moderate enhancement of the A_e_, based on the high surface-to-volume ratio of AuNPs, which slightly increase surface roughness and the number of electrochemically accessible sites [37, 38].

However, despite the observed slight increase in the A_e_ upon AuNPs-modification, a notable improvement in the electrochemical performance was demonstrated by DPV measurements of 50 µM SE (see Figure S5A). AuNPs-electrode (red signal) exhibits a better-defined electrochemical signal compared to the bare one (black signal), with a smoother peak shape, attributed to the enhanced electron transfer kinetics and the employment of a homogeneous AuNPs colloidal suspension. Besides, although the A_e_ increases moderately (ca. 20% *vs*. bare electrode), a much larger enhancement of the maximum peak current (35–40%) was observed. Despite the use of AuNPs is primarily motivated by the gold well-established thiol chemistry, that allows stable anchoring of the selected aptamers, this intrinsic improvement of the surface electrochemistry is a noteworthy outcome.

Then, fabrication stages of the aptasensor ((i) bare electrode, (ii) AuNPs-electrode, (iii) aptamers-AuNPs-electrode, and (iv) MCH-aptamers-AuNPs-electrode) were explored by EIS (Figure S5B). Except for the last stage, the impedance data were best fitted using a modified Nyquist equivalent circuit of the form R_1_[(R_2_Q)W], in which the ideal capacitive element (C) was replaced by a constant phase element (Q), reflecting the non-ideal capacitive behavior of the system. The data resulting from the fitting are summarized in Table S1. The charge transfer resistance (R_2_ = R_ct_) was effectively monitored at each step of the PEAs fabrication process, and its value reflected on the semicircle’s diameter of the Nyquist plots. Compared to the bare electrode (Figure S5B, black dots), the modification with AuNPs (AuNPs-electrode, Figure S5B, red dots) resulted in a significant decrease in R_ct_ from 859 to 54 Ω. This indicates that AuNPs enhance the electron transfer rate between the WE and the redox probe [Fe(CN)_6_]^3−/4−^, primarily due to its highly efficient electron transport properties and the gold layer’s large specific surface area. Following the assembly of thiolated aptamers onto the AuNPs-electrodes to obtain aptamers-AuNPs-electrodes (Figure S5B, blue dots), a larger semicircle was observed, and the R_ct_ value significantly increased to 1220 Ω. This increase is attributed to the negatively charged phosphate backbone of the immobilized aptamers, which repels the negatively charged [Fe(CN)_6_]^3−/4−^ redox probe [39]. This behavior is consistent with ζ‑potential measurements of the aptamers in solution, which exhibited a negative value of −12 ± 1.5 mV, confirming their net negative surface charge under the experimental conditions. Upon treatment with MCH (MCH-aptamers-AuNPs-electrode), the R_ct_ value is estimated to increase until reaching around 2000 Ω (Figure S5B, green dots), based on the interpolation of a semicircle equivalent to the one used in the fitting applied for the other measurements. The observed blocking effect explains the slower interfacial electron transfer kinetics of the redox probes. These results confirm the successful formation of a biorecognition layer capable of selectively binding to the target. The reported values are estimated using the same circuit; however, it must be noticed that the surface must exhibits clear heterogeneity, including island or domains that do not allow a good description of the process using Randles equivalent circuit. Thus, for the last step of the modification, only experimental data was plotted.

**Fig. 3:**
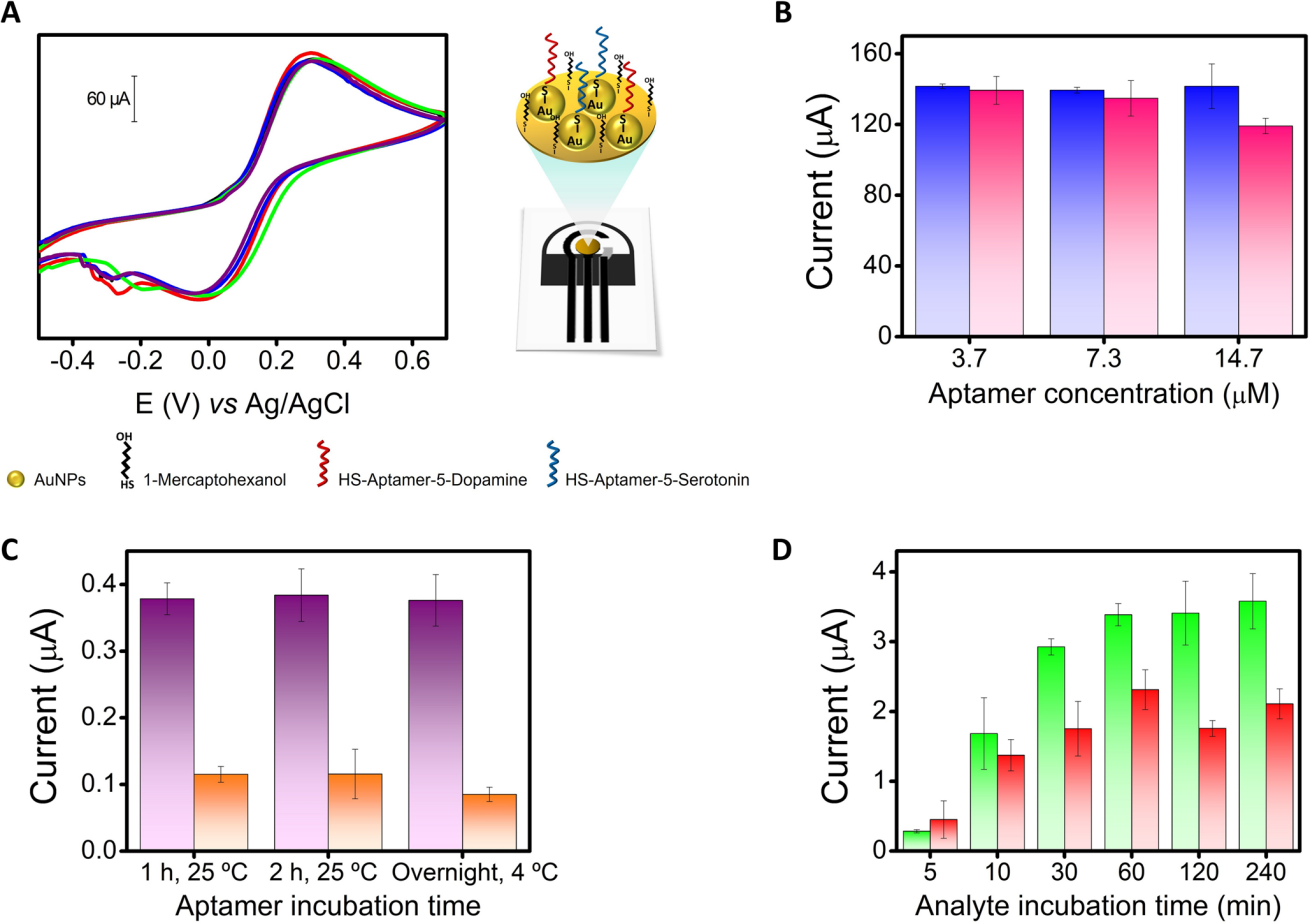
**(A)** CV of five PEAs under surface optimized conditions (black, red, blue, green, and purple lines) using 50 µL of 5 mM K_4_Fe(CN)_6_/K_3_Fe(CN)_6_ in 0.1 M KCl electrolyte as redox probe (left) and the schematics of the fabricated PEA (right). **(B)** Comparison between different aptamer concentration for each type of thiolated aptamer by CV using 50 µL of 5 mM K_4_Fe(CN)_6_/K_3_Fe(CN)_6_ in 0.1 M KCl electrolyte as redox probe. Thiolated aptamer of SE (blue bar) and thiolated aptamer of DA (pink bar) (*n* = 3 PEAs). CV parameters: start potential − 0.5 V, end potential + 1.0 V, scan rate 0.1 V s^− 1^. **(C)** Comparison between different incubation times of the thiolated aptamers by DPV using 10 µM SE or DA. Thiolated aptamer of SE (purple bar) and thiolated aptamer of DA (orange bar) (*n* = 3 PEAs). **(D)** Comparison between different incubation times for each aptamer type with its corresponding analyte by DPV using 50 µM SE or DA. SE (green bar) and DA (red bar). DPV parameters: see Fig. 2. Values are expressed as Mean Values ± S.D (*n* = 3 PEAs)

To further assess the quality and reproducibility of the electrode surface, CV measurements were performed using the [Fe(CN)_6_]^3−/4−^ redox probe on five fabricated PEAs under final optimized conditions (MCH-aptamers-AuNPs-electrode) (see below for optimization details). The CVs shown in Fig. 3A exhibited an excellent inter-PEA reproducibility, with an average anodic peak current of 0.135 ± 0.006 mA (RSD < 5%) and an average cathodic peak current of − 0.083 ± 0.004 mA (RSD < 5%) (*n* = 5 PEAs). Additionally, good electrochemical reversibility was also observed, with a mean peak-to-peak separation (ΔE_p_) of − 330 ± 12 mV (*n* = 5 PEAs). These results revealing the quality of the electroactive surfaces.

Then, aptamer concentration, aptamer incubation time, and analyte incubation time were carefully optimized (see Fig. 3B−D).

The optimization of aptamer concentration was first performed individually for each type of thiolated aptamer, and then using both aptamers simultaneously. Solutions of 3.7, 7.3, and 14.7 µM of thiolated SE and DA aptamers were tested individually by CV using 50 µL of 5 mM [Fe(CN)_6_]^3−/4−^ in 0.1 M KCl electrolyte as redox probe (see Figure S6 for representative CVs), as can be observed in the summarized Fig. 3B as bar plots. No significant differences were obtained between the three concentrations for each type of aptamer, so the concentration of 3.7 µM of thiolated SE and DA aptamers was selected (see Fig. 3B, blue and pink bars, respectively).

The incubation times of the thiolated aptamers for their immobilization on the gold electrode surface through the Au–thiol binding were then studied. Initially, the optimization of incubation times was performed individually for each type of thiolated aptamer, and then using both aptamers simultaneously. Different incubation times and temperatures were evaluated for each type of aptamer (Fig. 3C). After the incubation times with the thiolated aptamers, the electrode surface was washed to remove excess aptamer, then blocked with 15 µL of 7 mM MCH in PBS, subsequently, 15 µL of 10 µM SE or DA was dropped onto the WE and incubated for 1 h at room temperature. After this step, the surface was washed again and covered with 50 µL of PBS (0.1 M, pH 7.2) for electrochemical measurement by DPV. No significant differences were observed among the three incubation times and temperatures for each aptamer type, so 1 h at 25 °C was selected as the optimal incubation time over the surface of the AuNPs electrode for the thiolated aptamer of SE and the thiolated aptamer of DA, as it provided strong analytical signals in a reduced time (see Fig. 3C, purple and orange bars, respectively).

Next, different blocking agents (BSA, MCH, and 1,6-hexanedithiol, using 15 µL of blocking agent for 1 h) [40] were evaluated on the electrode surface to prevent nonspecific binding using the PEAs (*n* = 3 PEAs). MCH was chosen as the optimal blocking agent (data not shown) because it obtained the best analytical responses in terms of sensitivity and peak resolution. Once MCH was chosen as the optimal blocking agent, the next study consisted of optimizing the MCH concentration. Solutions of 1, 3, 7, 10, 80, and 100 mM MCH (in 0.1 M PBS pH 7.2) were tested, until dryness (1 h at room temperature), blocking the electrode surface with 15 µL of MCH solution using the PEAs (*n* = 3 PEAs). 7 mM MCH was chosen as the optimal concentration (results not shown) since a balance was achieved between background noise, sensitivity and resolution of the DA and SE peaks, and at that concentration, the MCH droplet was retained on the surface of the WE without leaking.

Finally, different incubation times were evaluated for each aptamer type with its corresponding analyte to balance response time and signal integrity (Fig. 3D). 15 µL of 50 µM analyte (SE or DA) were deposited on the WE and an incubation time of 5, 10, 30, 60, 120, and 240 min was waited. After that time, the surface was washed three times with 20 µL of PBS (0.1 M, pH 7.2) and the entire electrochemical cell was covered with 50 µL of PBS (0.1 M, pH 7.2) to carry out the electrochemical measurement by DPV. For SE, a significant increase in current intensity is observed up to 60 min, at which time the current intensity remains constant for longer times (see Fig. 3D, green bar). In the case of DA, a significant increase in current intensity was obtained up to 60 min, at which point the current intensity remained constant or slightly lower at longer times (see Fig. 3D, red bar). For these reasons, 60 min was chosen as the optimal incubation time for the analyte (or sample) with each aptamer type.

Table 1 provides a summary of the variables investigated, including their studied ranges and corresponding optimized values, as discussed above.

**Table 1:** PEA optimization for simultaneous and selective detection of DA y SE

Parameter	Investigated range	Optimized value
Aptamer concentration	3.7–14.7 µM	3.7 µM, each
Aptamer incubation time	1 h at 25 °C 2 h at 25 °C Overnight at 4 °C	1 h, 25 °C
Blocking agent	BSA MCH 1,6-hexanedithiol	MCH (data not shown)
MCH concentration	1–100 mM	7 mM (data not shown)
Analyte incubation time	5–240 min	60 min

BSA, bovine serum albumin; MCH 1-mercaptohexanol

### Analytical performance of the paper-based electrochemical aptasensor

**Fig. 4:**
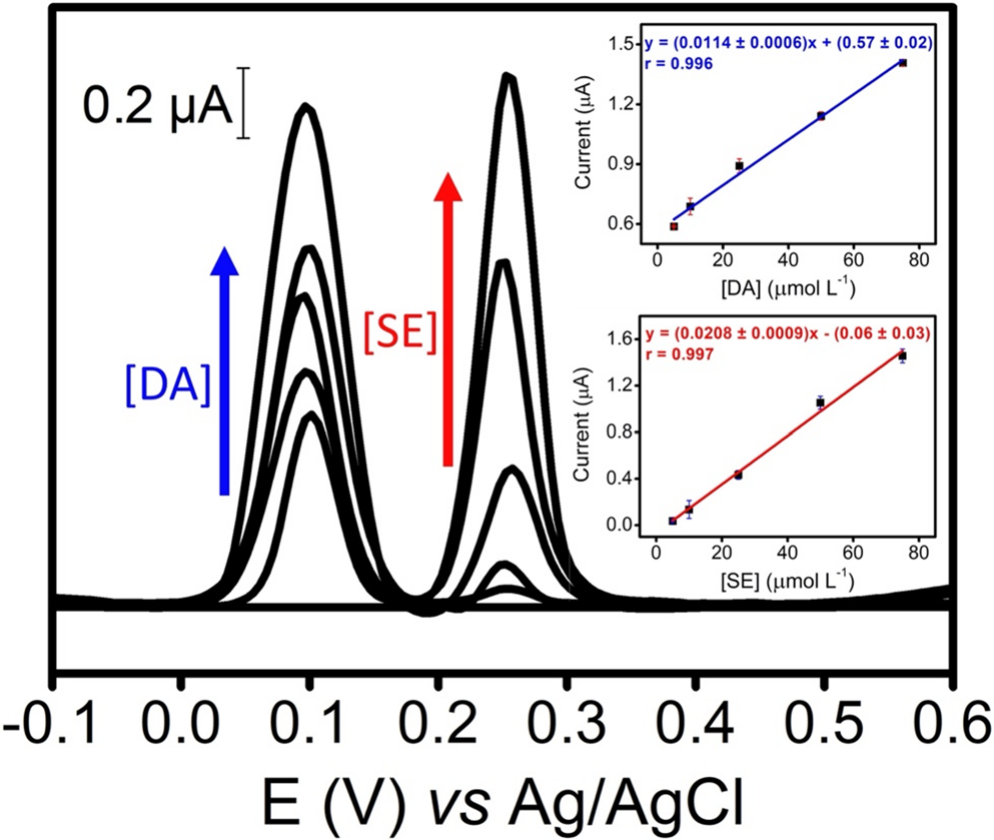
Voltammograms corresponding to a mixed of DA and SE standard solutions (0.1 M PBS, pH 7.2) from 5 to 75 µmol L^− 1^ using the PEAs. Insets: Calibration plot of current intensity *vs*. the concentration of DA (peak at + 0.10 V, blue line) and SE (peak at + 0.25 V, red line). The backgrounds signal was corrected. Each point on the calibration plots corresponds to the use of three independent disposable PEAs. Values are expressed as Mean Values ± S.D (*n* = 3 PEAs). For other DPV parameters: see Fig. 2

Figure 4 shows the linear calibration plots obtained simultaneously in a dual manner for the standard neurotransmitter mixtures of DA and SE by DPV. Each point on the calibration plots corresponds to the use of three independent disposable PEAs for dual neurotransmitter label-free detection.

The calibration curve for DA, constructed recording the voltammetry peak current at + 0.10 V (blue inset of Fig. 4), was linear in the range of 5–75 µmol L^− 1^, showing an excellent correlation coefficient (*r* = 0.996). The calibration slope was 0.0114 ± 0.0006 µA L µmol^− 1^ and the intercept was 0.57 ± 0.02 µA. In addition, the reproducibility of PEAs in terms of calibration slopes was very good (RSD = 5%). The limit of detection (LOD) was 0.80 µmol L^− 1^ (calculated as 3 s/b criteria, where s is the standard deviation of lowest calibration concentration (*n* = 3 PEAs) and b the slope of calibration plot). The calibration curve for SE, constructed recording the voltammetry peak current at + 0.25 V (red inset of Fig. 4), was linear in the range of 5–75 µmol L^− 1^, showing an excellent correlation coefficient (*r* = 0.997). The calibration slope was 0.0208 ± 0.0009 µA L µmol^− 1^ and the intercept was 0.06 ± 0.03 µA. The reproducibility of PEAs in terms of calibration slopes was very good (RSD = 4%). LOD was 1.30 µmol L^− 1^ (calculated as 3 s/b criteria, where s is the standard deviation of lowest calibration concentration (*n* = 3 PEAs) and b the slope of calibration plot). Overall, the robustness coupled to the easy manufacturing process of the PEAs has been highlighted.

### Assessment of extracellular levels of DA and SE in left prefrontal cortex tissue samples

**Fig. 5:**
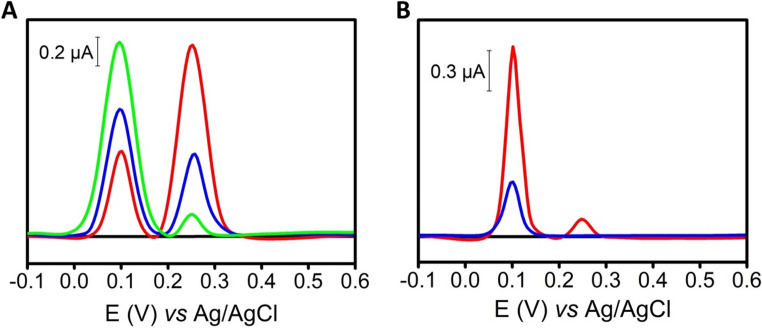
**(A)** Voltammograms obtained by DPV for sample AD-6 of extract from the left prefrontal cortex of a patient with AD, which shows no detectable DA or SE peaks (black line), and for the same sample spiked with 10 µM DA and 70 µM SE (red line), spiked with 30 µM DA and 30 µM SE (blue line), and spiked with 70 µM DA and 10 µM SE (green line), using the PEA. **(B)** Voltammograms obtained by DPV for blank (black line), sample H-2 (without AD, red line), and sample AD-1 (with AD, blue line) for the assessment of extracellular levels of DA and SE in samples of extracts of left prefrontal cortex tissue using the PEAs. Values are expressed as Mean Values ± S.D (*n* = 3 PEAs). DPV parameters: see Fig. 2 (the background signal was corrected)

DA and SE concentrations during AD progression remain poorly standardized and highly variable across the literature, particularly in complex brain matrices. To evaluate the analytical accuracy of the proposed PEAs under realistic conditions, recovery studies were performed using left prefrontal cortex tissue samples from an AD patient (AD-6) that did not exhibit detectable DA or SE oxidation peaks (Fig. 5A, black line). The undiluted sample was subsequently spiked with well-defined mixtures of DA and SE at three concentration levels (Fig. 5A, red, blue, and green lines, respectively), enabling assessment of method performance in a biologically relevant matrix.

Table 2 lists the quantitative and reproducible recoveries obtained for the assayed DA–SE mixtures, in which both neurotransmitters were simultaneously measured on the same WE, demonstrating excellent analytical accuracy for the quantification of DA and SE.

**Table 2:** DA and SE recoveries at three mixture concentration levels in a blank protein extract of left prefrontal cortex tissue (AD-6) by using the PEAs^1^

DA–SE mixture concentration		Added (µmol L^− 1^)	Found (µmol L^− 1^)	Recovery (%)
1	DA	10	9.4 ± 0.7	93 ± 7
	SE	70	70 ± 4	100 ± 6
2	DA	30	29 ± 1	95 ± 4
	SE	30	30 ± 2	99 ± 7
3	DA	70	70 ± 3	100 ± 5
	SE	10	10.2 ± 0.5	102 ± 4

^1^ Values are expressed as Mean Values ± S.D (n = 3 PEAs)

Next, since both neurotransmitters are known to play a key role in cognitive functions and are markedly reduced in AD [23, 28], to assess the applicability of the proposed PEAs in clinical samples, extracellular levels of DA and SE were measured in pseudonymized samples of protein extracts of the left prefrontal cortex tissue from individuals with and without AD. Figure 5B illustrates representative voltammograms obtained for the clinical samples, highlighting the differences between healthy individuals and AD patients samples. In contrast with the blank (black line), the DA and SE peaks of sample H-2 (healthy individuals, Fig. 5B, red line) were clearly identified with excellent S/N ratios, in agreement with the expected potentials for DA (+ 0.10 V) and SE (+ 0.25 V). Conversely, sample AD-1 (AD patients, Fig. 5B, blue line) exhibited a drastic decrease in the DA signal, and the absence of the SE signal compared to the healthy sample H-2, confirming the neurotransmitter depletion typically associated with AD.

**Fig. 6:**
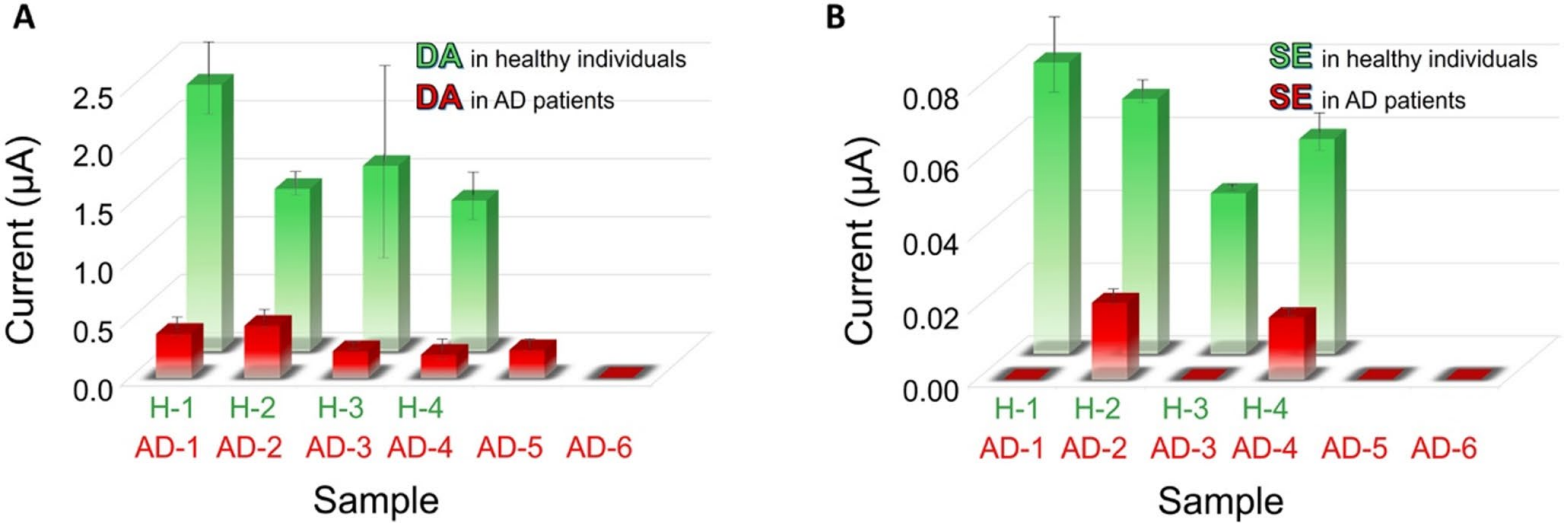
Analysis of extracellular levels of DA **(A)** and SE **(B)** in samples of extracts of left prefrontal cortex tissue from independent healthy individuals (green bars) and patients with AD (red bars) by PEAs. Values are expressed as Mean Values ± S.D. (*n* = 3 PEAs)

To provide a comprehensive view of the results, Fig. 6 and Table S2 summarize the peak height and concentration values, respectively, for DA and SE simultaneously measured in all pseudonymized samples from healthy individuals (H-1 – H-4) and AD patients (AD-1 – AD-6) using the PEAs approach.

As stated above, quantitatively, DA and SE concentrations during AD progression remain poorly standardized and highly variable across reports; however, a progressive decline is consistently observed. In this study, we found a marked decrease in both DA and SE concentrations from those found in healthy individuals to no detectable ones in AD samples (Table S2). It is pertinent to say that levels measured in healthy individuals were higher for DA than for SE, for which a measurable decrease was observed in only one of the analyzed samples. Nevertheless, depletion of both neurotransmitters was detected overall, revealing dopaminergic and serotonergic dysfunctions, which are easily noticed in the decreasing of electrochemical signals (Fig. 6). Indeed, as expected, both neurotransmitters exhibited significantly lower peak heights in AD samples compared to healthy controls (t-test, α = 0.05, two-sided, *n* = 3 PEAs), demonstrating the assay’s ability to reliably distinguish at the signal level between the two groups in this study.

Collectively, these findings confirm the PEAs capabilities to monitor biologically meaningful concentration shifts. Notably, the method operates across a clinically relevant dynamic range extending a decreasing of neurotransmitters levels from concentrations of healthy tissues to those observed in advanced pathology. This analytical window, combined with the intrinsic scalability and integrability of PEA architectures, supports their implementation in multiplexed biosensing in organ-on-chip platforms and highlights their potential translation toward liquid biopsy–based neurochemical monitoring. Again, it is important to note that physiological reference intervals for DA and SE in this specific biological matrix have not been firmly established. Therefore, the absolute concentrations reported here should not be interpreted against defined clinical thresholds but rather comparatively, emphasizing intergroup differences and relative concentration changes.

In light with these previous published works (see Table S3), most reported strategies for simultaneous neurotransmitter detection rely solely on intrinsic electrochemical selectivity, exploiting differences in oxidation potentials. Yet these methods are typically limited to synthetic or spiked samples and rarely perform robustly in complex biological matrices. Aptamer-based molecular recognition has emerged as a complementary approach, offering high affinity and chemical stability, also combined with electrochemical discrimination. However, truly integrated analysis in clinical samples remains scarce. Notably, a recent sweat-monitoring study achieves simultaneous neurotransmitter detection [41], but uses separate electrodes and substrates tailored for wearable applications—fundamentally different from the clinical and diagnostic context of this work.

In comparison with previous reports, our work introduces an ePAD supporting an integrated dual-selectivity approach that merges molecular specificity with potential-resolved electroactivity on a single paper-based WE-termed PEA, enabling direct detection of DA and SE in complex matrices such as brain tissue. The device operates with ultra-low sample volumes and supports anticipatory neurochemical monitoring, providing a versatile and cost-effective tool, even disposable, for the early assessment of neurotransmitter imbalances associated with AD. Moreover, this strategy is inherently compatible with advanced multiplexed designs, highlighting the potential of paper-based platforms for translational biosensing applications.

## Conclusion

Pioneer, we report a PEA-based platform enabling the direct assessment of extracellular DA and SE in human brain samples from AD patients using ePAD technology. By integrating this portable, low-cost technology with a dual-selectivity strategy—aptamer-mediated molecular recognition coupled to electrochemical discrimination—the PEA achieves label-free, simultaneous detection of both neurotransmitters on a single disposable electrode. This unified architecture minimizes analytical variability while maintaining high selectivity in complex biological matrices. The paper-based architecture inherently supports POC applicability due to its low material cost, disposability, and compatibility with miniaturized and decentralized testing formats. Beyond its analytical performance, the platform establishes a framework for accessible neurochemical profiling, enabling reliable discrimination between healthy and AD brain tissue. By capturing neurotransmitter depletion across a clinically meaningful concentration range, the technology lays the groundwork for early-stage neurochemical stratification and prospective diagnostic applications. Although validated in post-mortem samples from a limited cohort, the approach provides a scalable path toward minimally invasive testing strategies and integration into brain-on-chip systems, enabling dynamic neurochemical mapping in physiologically relevant models. Moreover, the scalable nature of the paper-based format also opens avenues for the development of multiplexed PEA arrays targeting additional AD‑related biomarkers, enabling broader neurochemical monitoring and supporting future drug‑response studies. Collectively, this work provides initial insights toward bridging fundamental neurobiology and translational diagnostics, highlighting the potential of ePAD for early AD research and monitoring.

## Supplementary Information

Below is the link to the electronic supplementary material.

Supplementary Material 1 (DOCX 6.39 MB) 

## Data Availability

The authors declare that the data supporting the findings of this study are available within the paper and its Supplementary Information files. Should any raw data files be needed in another format they are available from the corresponding author upon reasonable request.
