# Supplementary material for: Label-free paper-based electrochemical aptasensor with tunable selectivity for assessing neurotransmitter imbalance in Alzheimer’s disease
Source: Mikrochim Acta. 2026 May 7;193(6):375. doi: 10.1007/s00604-026-08100-9 (PMC13152916; doi:10.1007/s00604-026-08100-9)
Supplement: Supplementary file 1 — Supplementary Material 1 (DOCX 6.39 MB) [file 604_2026_8100_MOESM1_ESM.docx]

**SUPPORTING INFORMATION**

**Label-Free Paper-Based Electrochemical Aptasensor with Tunable Selectivity for Assessing Neurotransmitter Imbalance in Alzheimer’s Disease**

**Silvia Dortez ^a,†^, Miriam Chávez ^a, b,†^, Ana Montero-Calle ^c^, Rodrigo Barderas ^c, d^, Marta Pacheco ^a**^, Alberto Escarpa ^a, e*^**

^a^ Department of Analytical Chemistry, Physical Chemistry and Chemical Engineering, University of Alcala, 28802, Alcala de Henares, Madrid, Spain.

^b^ Department of Physical Chemistry and Applied Thermodynamics, Institute of Chemistry for Energy and Environment, University of Cordoba, Campus Rabanales, Ed. Marie Curie, E-14014 Córdoba, Spain

^c^ Chronic Disease Programme, UFIEC, Institute of Health Carlos III, Majadahonda, 28220 Madrid, Spain

^d^ CIBER of Frailty and Healthy Aging, CIBERFES, 28029, Madrid, Spain.

^e^ Chemical Research Institute “Andrés M. Del Río” (IQAR), University of Alcala, 28802 Alcala de Henares, Madrid, Spain.

**Corresponding authors:**

*Alberto Escarpa, Department of Analytical Chemistry, Physical Chemistry and Chemical Engineering and Chemical Research Institute “Andres M. Del Rio”, Universidad de Alcalá, Madrid, Spain ([alberto.escarpa@uah.es](mailto:alberto.escarpa@uah.es))

**Marta Pacheco, Department of Analytical Chemistry, Physical Chemistry and Chemical, Universidad de Alcalá, Madrid, Spain ([marta.pacheco@uah.es](mailto:marta.pacheco@uah.es))

† S.D. and M.C contributed equally to this paper

**ORCID number:**

Silvia Dortez: 0000-0002-1999-5605

Miriam Chávez: 0000-0002-5512-7485

Ana Montero-Calle: 0000-0001-5141-0454

Rodrigo Barderas: 0000-0003-3539-7469

Marta Pacheco: 0000-0001-9546-9108

Alberto Escarpa: 0000-0002-7302-0948

**TABLE OF CONTENTS**

|  | **Pg.** |
| --- | --- |
| **ABBREVIATIONS** | S3 |
|  |  |
| **SUPPORTING FIGURES** |  |
| **Figure S1.** Secondary structure and thermodynamic parameters of SE and DA aptamers modeled with MFold software | S4 |
| **Figure S2.** Schematic representation of the fabrication of the ePAD | S5 |
| **Figure S3.** Dimensions of the ePAD | S6 |
| **Figure S4.** TEM images of pristine AuNPs and AuNP size by different techniques | S6 |
| **Figure S5.** Electrochemical performance of the ePAD using DPV and EIS | S7 |
| **Figure S6.** CVs corresponding to the thiolated aptamer of SE and DA using different aptamer concentration | S7 |
|  |  |
| **SUPPORTING TABLES** |  |
| **Table S1.** Quantitative evaluation of surface conductivity and electroactive area | S8 |
| **Table S2.** Analysis of extracellular levels of DA and SE in samples of extracts of the left prefrontal cortex brain tissue from individuals with and without AD by PEAs | S8 |
| **Table S3.** Electrochemical sensors for simultaneous DA and SE determination | S9 |
| **REFERENCES** | S10 |

**ABBREVIATIONS**

**AA**, ascorbic acid; **AD**, Alzheimer's disease; **A_e_**, electroactive surface area; **AuNPs**, gold nanoparticles; **BSA**, bovine serum albumin; **CV**, cyclic voltammetry; **DA**, dopamine; **DLS**, dynamic light scattering; **DPV**, differential pulse voltammetry; **EIS**, electrochemical impedance spectroscopy; **ePAD**, electrochemical paper-based analytical device; **Gluc**, Glucose; **LOD**, detection limits; **MCH**, 1-mercaptohexanol; **NE**, norepinephrine; **PBS**, Phosphate buffered saline; **PEA**, paper-based electrochemical aptasensor; **POC**, *point-of-care*; **RSD**, relative standard deviation; **SE**, serotonin; **TEM**, transmission electron microscopy; **UA**, uric acid; **UV–vis**, ultraviolet–visible; **WE**, working electrode.

**SUPPORTING FIGURES**


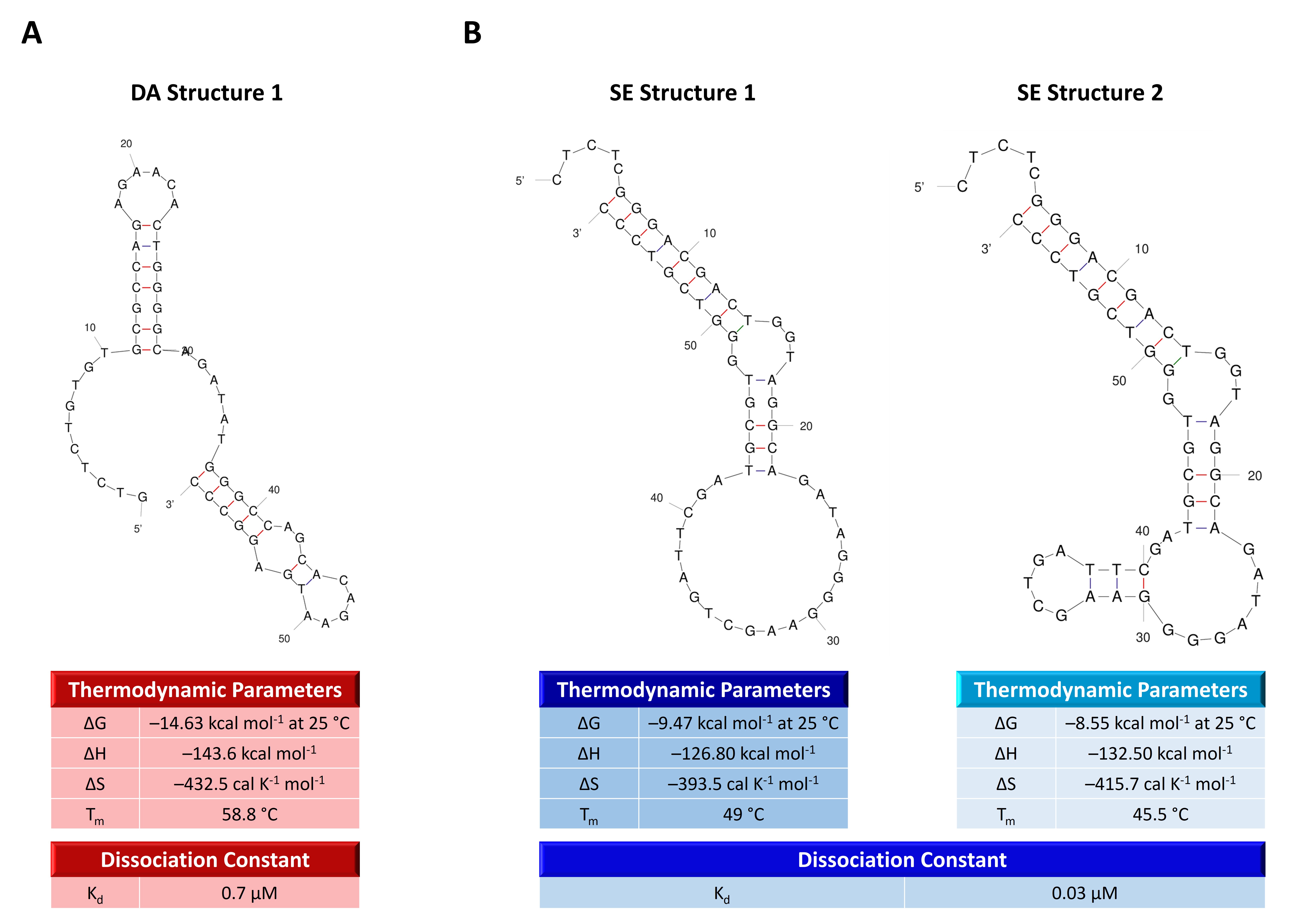


**Figure S1.** Dissociation constant [1, 2], secondary structure, and thermodynamic parameters of DA **(A)** and SE **(B)** aptamers modeled with MFold software.





**Figure S2.** Schematic representation of the fabrication of the ePADs: drawing, market and cutter plot, stencil-printing, sealed with clear packing tape, and drop casting.


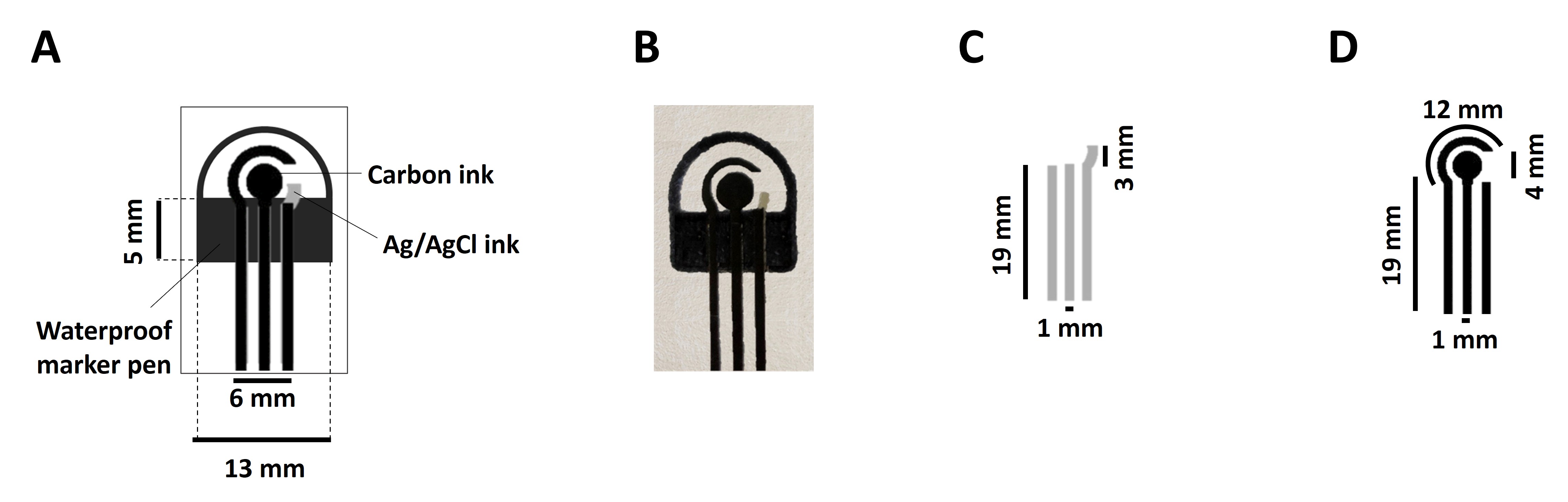


**Figure S3. (A)** Dimensions of the ePADs for label-free multiplex electrochemical detection of SE and DA. **(B)** Image of an ePAD. **(C)** Ag/AgCl ink pattern. **(D)** Carbon ink pattern.


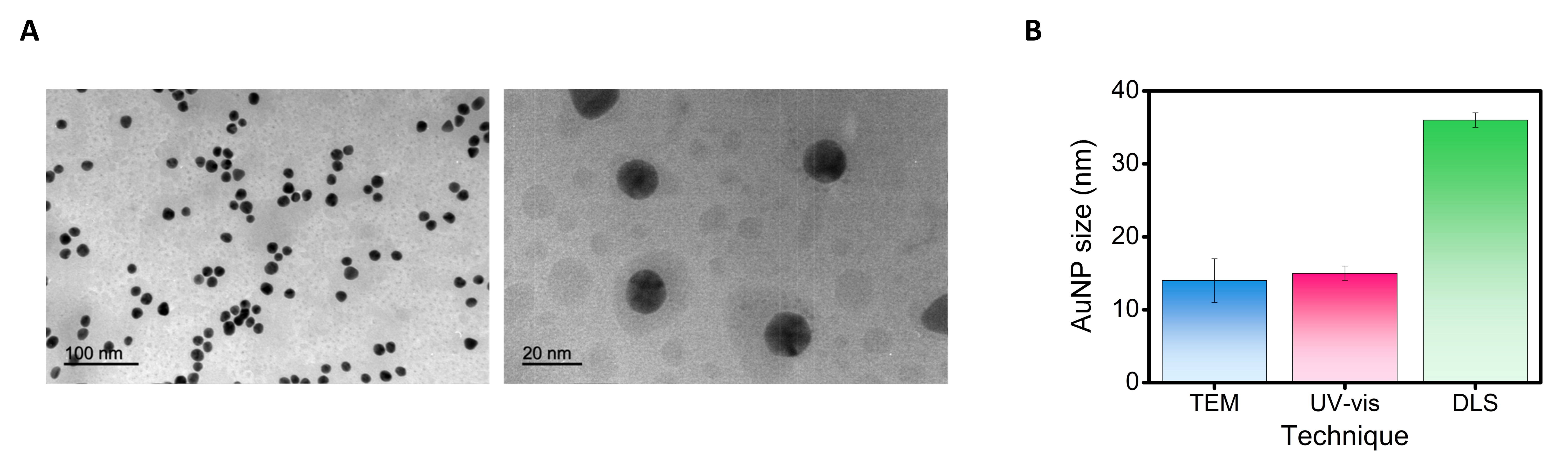


**Figure S4. (A)** TEM images of pristine AuNPs: low-magnification demonstrates the homogeneous distribution of nanoparticles (scale bar: 100 nm, left) and high-resolution image of quasi-spherical individual particles (scale bar: 20 nm, right). **(B)** Core diameter of AuNPs by different techniques: TEM (blue bar), UV–vis (pink bar), and DLS (green bar). Values are expressed as Mean Values ± S.D. (n = 3).


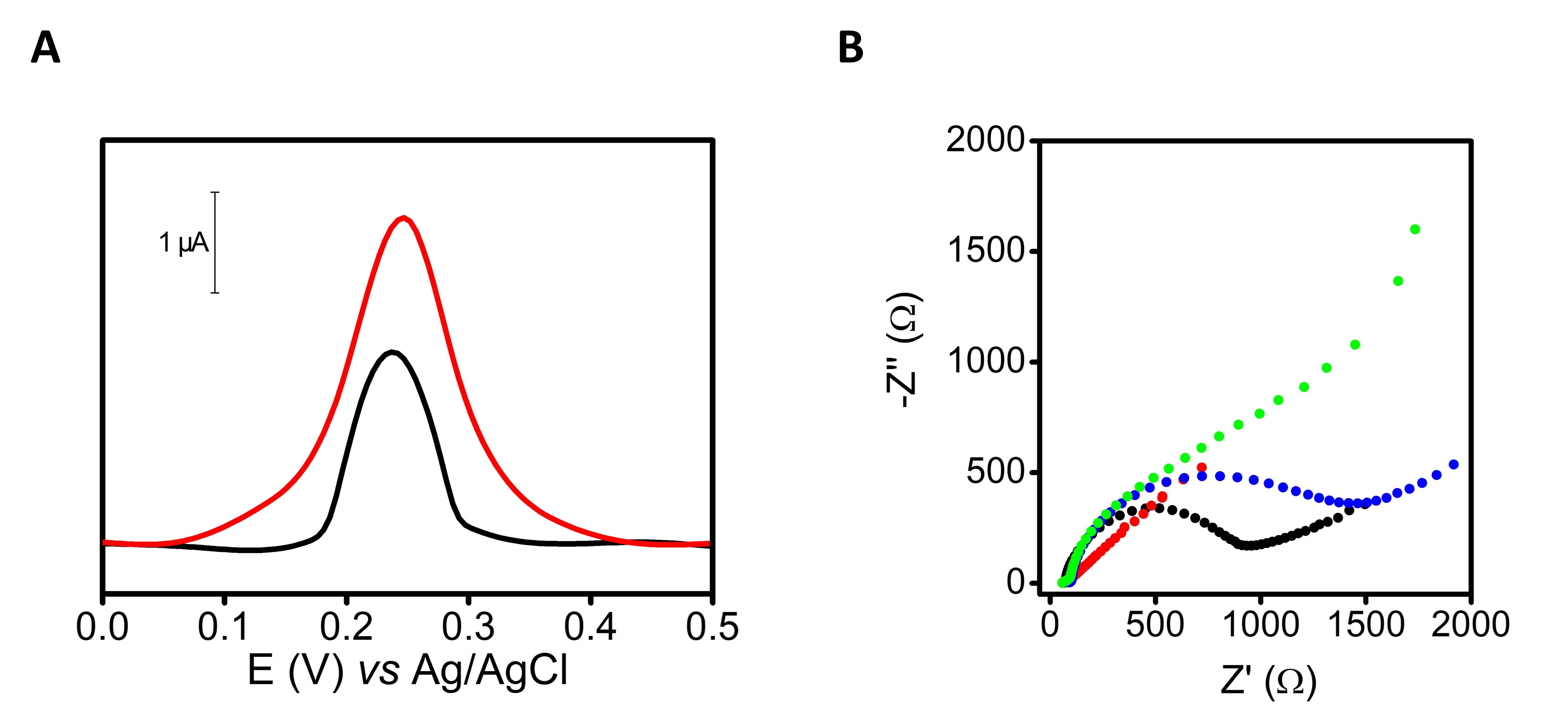


**Figure S5. (A)** DPV measurements of 50 μM SE (PBS 0.1 M, pH 7.2) recorded using a bare electrode (black line), and after its modification via drop casting with 10 nM AuNPs-colloidal suspension (red line). DPV parameters: start potential –0.1 V, end potential +0.6 V, step potential 0.005 V, amplitude 0.025 V, and modulation time 0.05 s (the background signal was corrected) (n = 3 PEAs). **(B)** Nyquist plots obtained by EIS with 50 µL of 5 mM K_4_Fe(CN)_6_/K_3_Fe(CN)_6_ in 0.1 M KCl electrolyte as redox probe for the bare electrode (black dots), AuNPs-electrode (red dots), aptamers-AuNPs-electrode (blue dots), and MCH-aptamers-AuNPs-electrode (green dots). EIS parameters: E_ap_ = E_1/2_ = 0.1 V, f_range_= 10^5^ – 0.01 Hz (n = 3 PEAs).


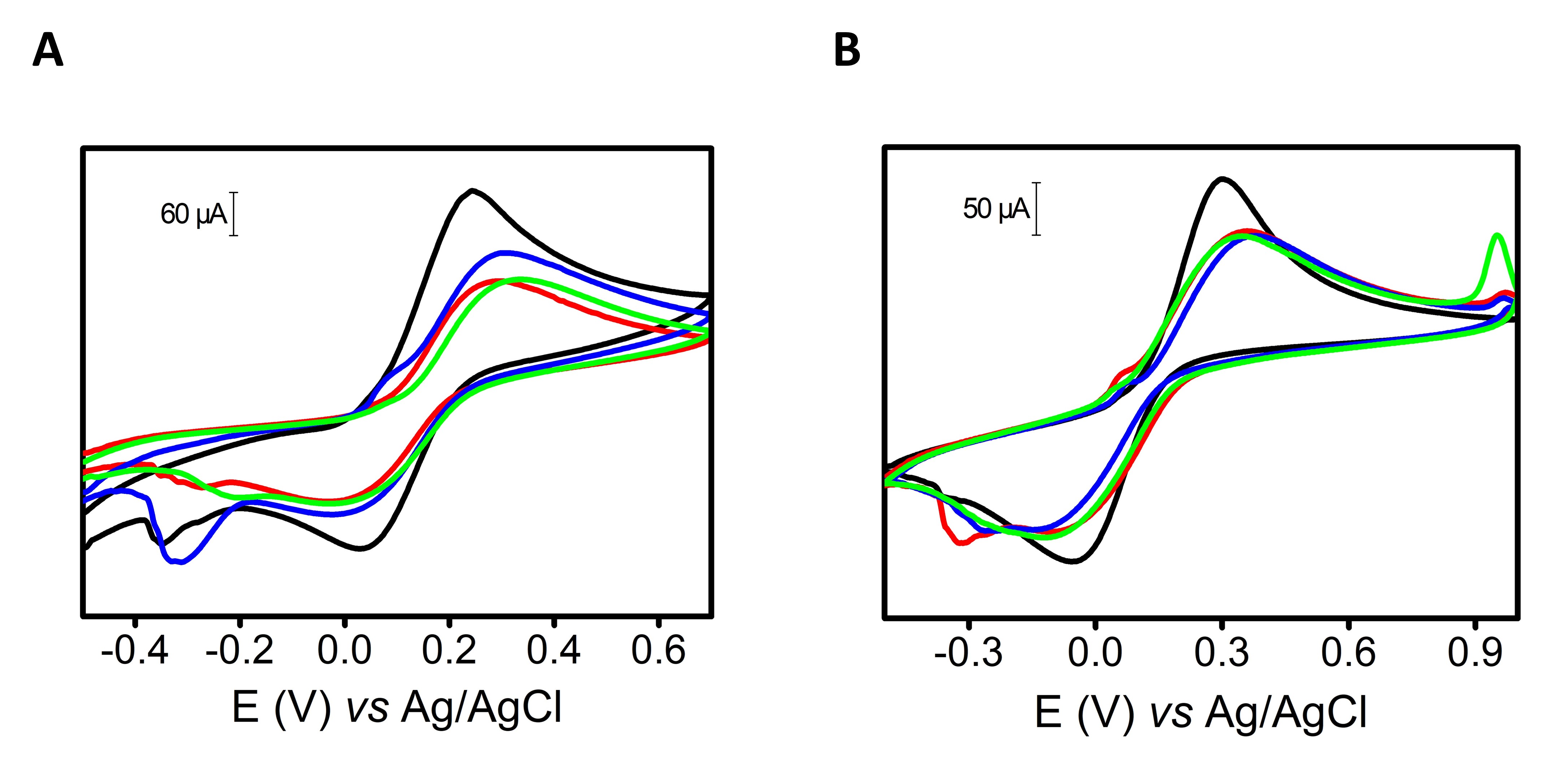


**Figure S6. (A)** CV of AuNPs-electrodes (black line), SE Apt (3.7 µM)-AuNPs-electrode (red line), SE Apt (7.3 µM)-AuNPs-electrode (blue line), and SE Apt (14.7 µM)-electrode-ePAD (green line) using 50 µL of 5 mM K_4_Fe(CN)_6_/K_3_Fe(CN)_6_ in 0.1 M KCl electrolyte as redox probe. **(B)** CV of AuNPs-electrode (black line), DA Apt (3.7 µM)-AuNPs-electrode (red line), DA Apt (7.3 µM)-AuNPs-electrode (blue line), and DA Apt (14.7 µM)-AuNPs-electrode (green line) using 50 µL of 5 mM K_4_Fe(CN)_6_/K_3_Fe(CN)_6_ in 0.1 M KCl electrolyte as redox probe. CV parameters: start potential –0.5 V, end potential +1.0 V, scan rate 0.1 V s^-1^ (n = 3 PEAs).

**SUPPORTING TABLES**

**Table S1.** Quantitative evaluation of surface conductivity and electroactive area enhancement achieved through the proposed fabrication strategy using CV and EIS in the presence of the [Fe(CN)₆]³⁻^/^⁴⁻ redox probe^1^.

| **Electrode** | **R_1_ (Ω)** | **R_2_ (Ω)** | **Q (μMho·sⁿ)** | **n** | **W (mMho·s^1/2^)** | **C (mF·cm^-2^)** |
| --- | --- | --- | --- | --- | --- | --- |
| Bare electrode | 69 ± 4 | 859 ± 7 | 7 ± 10 | 0.84 ± 0.02 | 1.84 ± 0.10 | 0.27 ± 0.02 |
| AuNPs-electrode | 53 ± 3 | 54 ± 4 | 68 ± 10 | 0.4 ± 0.1 | 0.88 ± 0.03 | 755 ± 38 |
| Aptamers-AuNPs-electrode | 94 ± 4 | 1220 ± 12 | 23 ± 10 | 0.84 ± 0.02 | 1.53 ± 0.06 | 2.7 ± 0.1 |

^1^ Values are expressed as Mean Values ± S.D. (n = 3 PEAs).

**Table S2**. Analysis of extracellular levels of DA and SE in samples of extracts of the left prefrontal cortex brain tissue from individuals with and without AD by PEAs^1^.

| **Sample** | **DA (μM)** | **SE (μM)** |
| --- | --- | --- |
| H-1 | > ULLQ^2^ | 1.4 ± 0.4 |
| H-2 | 72 ± 13 | < LOD |
| H-3 | > ULLQ^2^ | < LOD |
| H-4 | 67 ± 21 | < LOD |
| AD-(1–6) | < LOD | < LOD |

^1^ Values are expressed as Mean Values ± S.D. (n = 3 PEAs).

^2^ ULLQ: Upper Limit of Linear Quantification.

**Table S3.** Electrochemical sensors for simultaneous DA and SE determination.

|  | **Electrochemical technique** | **Electrode** | **LOD** | **Sample** | **Remarks** | **Ref.** |
| --- | --- | --- | --- | --- | --- | --- |
| **I** | DPV | Glassy carbon electrode/Pt‑doped reduced graphene oxide | 0.5 µM DA  0.4 µM SE | Spiked human urine and serum | Electroactive-based selectivity  Nanomaterial enhanced sensitivity | [3] |
|  | SVW | ITO electrode/sulfonated carbon nanoparticles encapsulated in silica | 0.10 µM DA  0.01 µM SE | Spiked mice blood serum (70 µL) | Electroactive-based selectivity  Interference‑free *vs* UA/AA | [4] |
|  | DPV | Pencil graphite electrode/ AuNPs/ poly(L‑lysine)/graphene quantum dots | 0.03 µM DA  0.017 µM SE | Spiked and diluted fetal bovine serum | Electroactive-based selectivity  Conductive nanomaterials improving sensitivity | [5] |
|  | DPV | 16 Au microelectrodes/rGO/PEDOT:PSS/Nafion integrated on flexible polyimide probe | 0.5 µM DA  0.4 µM SE | Buffer solution | Electroactive-based selectivity  Multi-electrode array  No sample analysis | [6] |
|  | SWV | Glassy carbon electrode/chitosan–alginate polyelectrolyte complex + electrochemically reduced graphene oxide | 4.18 nM DA  3.23 nM SE | Spiked synthetic urine | Electroactive-based selectivity  Very low LODs for DA and SE | [7] |
|  | Amperometry | Boron‑doped diamond disk electrode | 0.2 µM DA  0.8 µM SE | Neuron cultivation media | Electroactive-based selectivity  Biofouling-resistant | [8] |
|  | SWV / CV | Au/Pt nano‑structured electrodes integrated on polymer nanostructured microchip | 0.12 µM DA  0.14 µM SE | N27‑A cells  (40 – 60 µL) | Electroactive-based selectivity  Static and dynamic (microfluidic) detection | [9] |
| **II** | DPV | Gold screen‑printed electrodes | 0.06 µM DA  0.12 µM SE | Spiked commercial serum samples | Integrated aptamer- electrochemical dual-selectivity  One working electrode & label-free detection approach  High diluted samples | [10] |
|  | SWV | Gold microelectrode array | 0.1 µM DA  0.5 µM SE | Buffer solution | Integrated aptamer- electrochemical dual-selectivity Multi‑electrodes & label-free detection approach  No sample analysis | [11] |
|  | SWV/  Amperometry | Flexible screen‑printed electrode modified with AuNPs–CuMOF@InMOF heterostructure and thiolated aptamers | 0.18 nM DA  0.33 nM SE | On‑body  human sweat | Integrated aptamer-electrochemical dual-selectivity Multi‑electrode patch & label-free detection approach  Microfluidic wearable sweat collection  MOFs for high surface area + enhanced electron transfer | [12] |
|  | DPV | Paper electrode modified with carbon and AuNPs | 0.80 µM DA  1.30 µM SE | Brain tissue extracts (15 µL) | Integrated aptamer- electrochemical dual-selectivity  One working electrode & label-free detection approach  Very low sample volumes  Highly versatile with multiplexed capabilities  Low-cost disposable approach for high potential in real time neurotransmitters monitoring in brain-on-chip systems. | This work |

**(I)** Electroactive-based selectivity, (**II)** Integrated electrochemical-aptamer dual-selectivity.

**REFERENCES**

1. Walsh R, DeRosa MC (2009) Retention of function in the DNA homolog of the RNA dopamine aptamer. Biochem Biophys Res Commun 388:732–735. https://doi.org/10.1016/j.bbrc.2009.08.084

2. Nakatsuka N, Yang K-A, Abendroth JM, et al (2018) Aptamer-field-effect transistors overcome Debye length limitations for small-molecule sensing. Science 362:319–324. https://doi.org/10.1126/science.aao6750

3. Elshafie H, Alqahtani AS, Mubarakali A, et al (2025) Simultaneous Electrochemical Detection of DA and 5-HT Using Pt-Doped-rGO Nanocomposite. J Clust Sci 36:77. https://doi.org/10.1007/s10876-025-02796-0

4. Kundys-Siedlecka M, Bączyńska E, Jönsson-Niedziółka M (2019) Electrochemical Detection of Dopamine and Serotonin in the Presence of Interferences in a Rotating Droplet System. Anal Chem 91:10908–10913. https://doi.org/10.1021/acs.analchem.9b02967

5. Şimşek N, Tığ GA (2022) Graphene Quantum Dot-poly(L-lysine)-gold Nanoparticles Nanocomposite for Electrochemical Determination of Dopamine and Serotonin. Electroanalysis 34:61–73. https://doi.org/10.1002/elan.202100442

6. Cha HB, Zhang Y, Yu H-Y, Lee YJ (2024) Flexible sensing probe for the simultaneous monitoring of neurotransmitters imbalance. Micro Nano Syst Lett 12:21. https://doi.org/10.1186/s40486-024-00211-3

7. Postolović KS, Radovanović MB, Stanić ZD (2025) Simultaneous determination of serotonin, dopamine, and ascorbic acid at a glassy carbon electrode modified with chitosan-alginate hydrogel and reduced graphene oxide. J Electroanal Chem 980:118992. https://doi.org/10.1016/j.jelechem.2025.118992

8. Lytvynenko A, Baluchová S, Zima J, et al (2024) Biofouling and performance of boron-doped diamond electrodes for detection of dopamine and serotonin in neuron cultivation media. Bioelectrochemistry 158:108713. https://doi.org/10.1016/j.bioelechem.2024.108713

9. Rubby MF, Fonder C, Uchayash S, et al (2024) *In situ* monitoring of neurotransmitters using a polymer nanostructured electrochemical sensing microchip. Microchem J 204:111159. https://doi.org/10.1016/j.microc.2024.111159

10. Cuhadar SN, Durmaz H, Yildirim-Tirgil N (2024) Multi-detection of seratonin and dopamine based on an electrochemical aptasensor. Chem Pap 78:7175–7185. https://doi.org/10.1007/s11696-024-03598-w

11. Sen D, Lazenby RA (2023) Selective Aptamer Modification of Au Surfaces in a Microelectrode Sensor Array for Simultaneous Detection of Multiple Analytes. Anal Chem 95:6828–6835. https://doi.org/10.1021/acs.analchem.2c05335

12. Fredj Z, Marvi F, Ullah F, Sawan M (2025) A wearable electrochemical aptasensor based MOF on MOF heterostructure for multi-neurotransmitters monitoring. Microchim Acta 192:384. https://doi.org/10.1007/s00604-025-07219-5
